# Investigation of a novel cilia-related gene *K04F10.2/KIAA0556* in *C. elegans*

**DOI:** 10.1186/2046-2530-1-S1-P43

**Published:** 2012-11-16

**Authors:** A Sanders, S Cevik, K Kida, R Bowie, O Blacque

**Affiliations:** 1 University College Dublin, Ireland

## 

Multiple proteomics and genomics approaches have been used to identify the molecular parts list of cilia and flagella. However, the specific cilia-related functions of many of these components remain unknown. Previously, *K04F10.2* was identified as a candidate cilia-related gene in *C. elegans*, exhibiting specific expression in ciliated sensory neurons. We now show that GFP-tagged *K04F10.2* is highly enriched at the transition zone (TZ) compartment at the base of the ciliary axoneme, and possibly to the more proximal transition fiber/basal body region. Fluorescence microscopy and transmission electron microscopy show that *K04F10.2* null mutants possess grossly normal cilium structure and ultrastructure. In contrast, wild-type worms overexpressing a *K04F10.2::gfp* transgene possess cilium integrity defects such as a dye-filling abnormality (Dyf) and phasmid cilia that are frequently short and abnormally separated. In addition, phasmid neuronal dendrites are abnormally short in these worms. Since GFP-tagged K04F10.2 is enriched at the TZ, we examined possible genetic relationships with known TZ-associated ciliary disease genes such as those causing Meckel-Gruber syndrome (MKS) and Nephronophthisis (NPHP). Unlike the synthetic Dyf (SynDyf) phenotypes known for alleles of various MKS and NPHP genes, no such phenotype was observed in *K04F10.2;mks-5* and *K04F10.2;nphp-4* double mutants. However, K04F10.2 was found to synthetically interact (SynDyf) with Joubert syndrome-associated Arl13b/arl-13, which interestingly does not localise at the TZ. Together, these data implicate K04F10.2 as a novel TZ-enriched protein with functions that are distinct from canonical MKS and NPHP module components.

